# H_2_S Donor, S-Propargyl-Cysteine, Increases CSE in SGC-7901 and Cancer-Induced Mice: Evidence for a Novel Anti-Cancer Effect of Endogenous H_2_S?

**DOI:** 10.1371/journal.pone.0020525

**Published:** 2011-06-27

**Authors:** Kaium MA, Yan Liu, Qing Zhu, Chun-hua Liu, Jun-Li Duan, Benny K-H. Tan, Yi Zhun Zhu

**Affiliations:** 1 Department of Pharmacology, School of Pharmacy and Institute of Biomedical Sciences, Fudan University, Shanghai, China; 2 Department of Cardiovascular Medicine, Ruijin Hospital of Shanghai Jiaotong University, Shanghai, China; 3 Department of Gerontology, Xinhua Hospital, Shanghai Jiaotong University, Shanghai, China; 4 Department of Pharmacology, Yong Loo Lin School of Medicine, National University of Singapore, Singapore, Singapore; University of Pennsylvania, United States of America

## Abstract

**Background:**

S-propargyl-cysteine (SPRC), an H_2_S donor, is a structural analogue of S-allycysteine (SAC). It was investigated for its potential anti-cancer effect on SGC-7901 gastric cancer cells and the possible mechanisms that may be involved.

**Methods and Findings:**

SPRC treatment significantly decreased cell viability, suppressed the proliferation and migration of SPRC-7901 gastric cancer cells, was pro-apoptotic as well as caused cell cycle arrest at the G_1_/S phase. In an *in vivo* study, intra-peritoneal injection of 50 mg/kg and 100 mg/kg of SPRC significantly reduced tumor weights and tumor volumes of gastric cancer implants in nude mice, with a tumor growth inhibition rate of 40–75%. SPRC also induced a pro-apoptotic effect in cancer tissues and elevated the expressions of p53 and Bax in tumors and cells. SPRC treatment also increased protein expression of cystathione-γ-lyase (CSE) in cells and tumors, and elevated H_2_S levels in cell culture media, plasma and tumoral CSE activity of gastric cancer-induced nude mice by 2, 2.3 and 1.4 fold, respectively. Most of the anti-cancer functions of SPRC on cells and tumors were significantly suppressed by PAG, an inhibitor of CSE activity.

**Conclusions:**

Taken together, the results of our study provide insights into a novel anti-cancer effect of H_2_S as well as of SPRC on gastric cancer through inducing the activity of a new target, CSE.

## Introduction

S-propargyl-cysteine (SPRC) is a structural analogue of S-allycysteine (SAC) with the same cysteine-containing structure. SAC, one of the major compounds in aged garlic extract, is derived from the degradation of *S*-alk(en)yl cysteine sulfoxides (ACSs). This compound has anti-tumor [1] [2], anti-bacterial [3], anti-fungal [4], anti-hepatotoxic [5], cardioprotective properties [6] and apoptosis [7]. Paclitaxel liposome, a lipid-based formulation of the anticancer drug, paclitaxel, has become a first-line drug for the treatment of refractory ovarian, breast, stomach and non-small cell lung cancers. We therefore investigated the *in vitro* and *in vivo* anticancer effects of SPRC and compared these with those of SAC and Paclitaxel liposome.

Cystathione-γ-lyase (CSE) is a member of the trans-sulfuration enzyme family and is responsible for catalyzing the pyridoxal phosphate-dependent β-disulfide elimination reaction resulting in ammonium, pyruvate and thiocysteine. Thiocysteine may then react with other thiols to generate H_2_S. The p53 tumor suppressor protein plays a major role in cellular response to DNA damage and other genomic aberrations. Activation of p53 can lead to either cell cycle arrest and DNA repair or apoptosis. Bax is a key component in cellular apoptosis through mitochondrial stress while Bcl-2 exerts a survival function in response to a wide range of apoptotic stimuli through inhibition of mitochondrial cytochrome c release. Here for the first time, we found that the anticancer effects of SPRC involved a hydrogen sulfide (H_2_S)-mediated pathway. SPRC can undergo β-elimination by CSE to produce endogenous H_2_S, which may up-regulate gene expression of p53 and Bax, resulting in suppression of cell proliferation and apoptosis. Our study thus showed a novel anti-cancer effect of the endogenous H_2_S donor, SPRC, on gastric cancer through inducing the activity of a new target, CSE. We demonstrated for the first time that SPRC was able to suppress cell proliferation and migration and also tumor growth in gastric cancer-induced model of nude mice, indicating that it may be a potential agent for the treatment of gastric cancer in humans. Our results showed that the anti-cancer effects of SPRC were attributed to suppression of cell cycle and induction of apoptosis. Furthermore, our results also showed that SPRC could regulate the gene expressions of CSE, Bax and p53. We found that PAG, an inhibitor of CSE activity, could significantly suppress the anticancer effects of SPRC.

## Results

### Dose-dependent, growth inhibitory and detection of the cytostatic effects of SPRC on SGC-7901 cells

**Figure 1:**
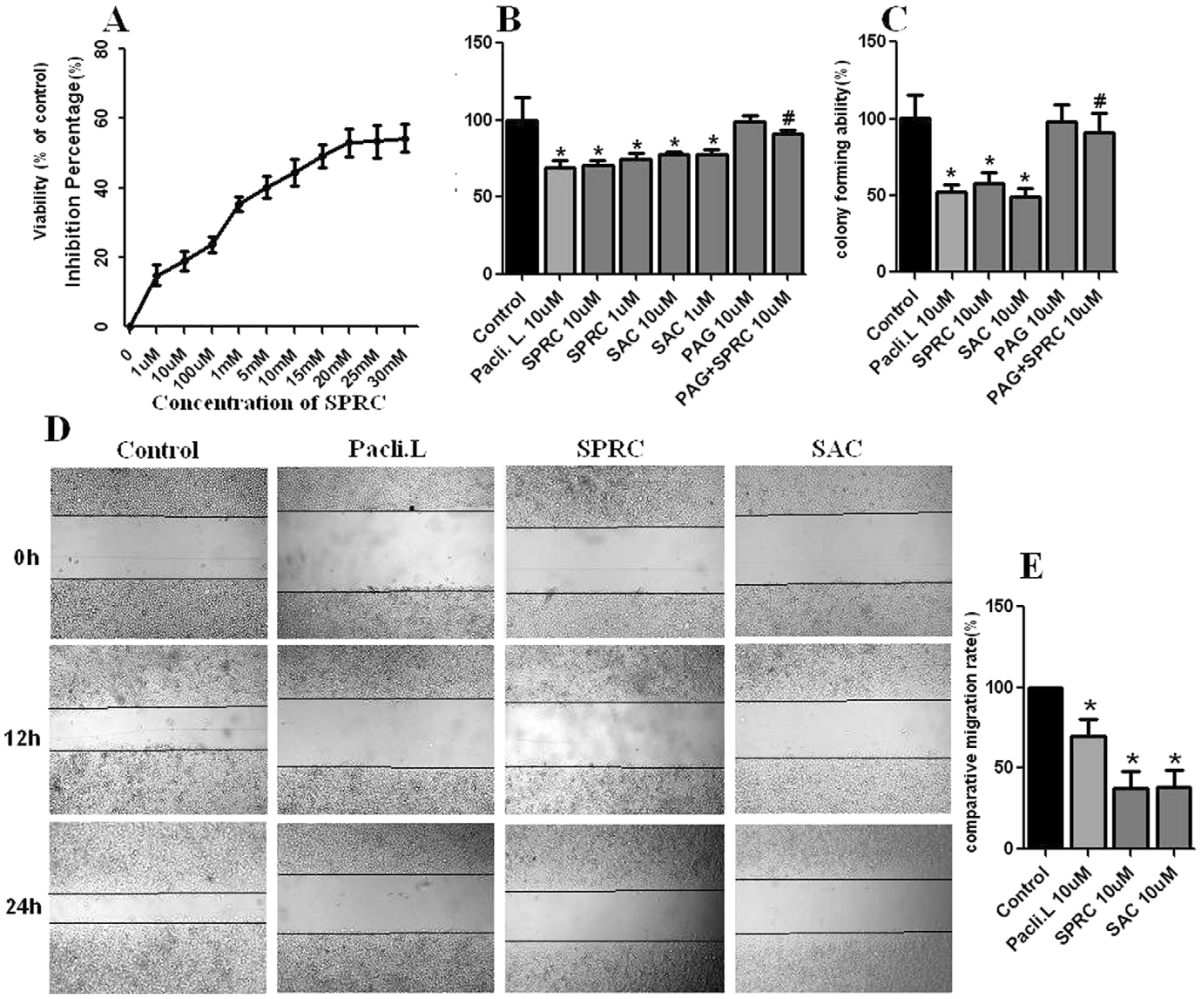
Growth inhibition of SGC-7901 cells under SPRC treatment. **A.** Dose-response study of the effects of SPRC on growth inhibition of SGC-7901 cells. **B.** Effects of SPRC, SAC, PAG, PAG+SPRC and paclitaxel liposome on viability of SGC-7901 cells. **C.** Effects of SPRC, SAC, PAG, PAG+SPRC and paclitaxel liposome on colony formation in SGC-7901 cells. **D.** Effects of SPRC, SAC and paclitaxel liposome on migration ability of SGC-7901 cells. Differential cell migration ability was examined by the wound-closure assay. **E.** Effects of SPRC, SAC and paclitaxel liposome on wound closure speed. Values are expressed as % of control. * represent significant difference between control vs. SPRC, SAC and paclitaxel liposome groups (p<0.01). ^#^ represent significant difference (p<0.01) between SPRC vs SPRC+PAG group (p<0.01).

Effects of SPRC on SGC-7901 gastric cancer cells were shown in Figure 1A, 1 uM and 10 uM SPRC produced 18–25% inhibition of viability of SGC-7901 cells. 20 mM to 30 mM SPRC caused about 50% cell viability inhibition. We also found that SPRC at low concentrations of 1 uM and 10 uM could significantly suppress the colony forming and migration ability of SGC-7901; these effects were more significant compared with SAC (Figures 1B, 1C and 1D). PAG, an inhibitor of CSE, blocked the inhibitory effect of SPCR on cell growth (Figures 1B and 1C). These results indicated that the CSE pathway was involved in SPRC-induced suppression of cell growth.

### Morphological changes, apoptosis and cell cycle analysis

**Figure 2:**
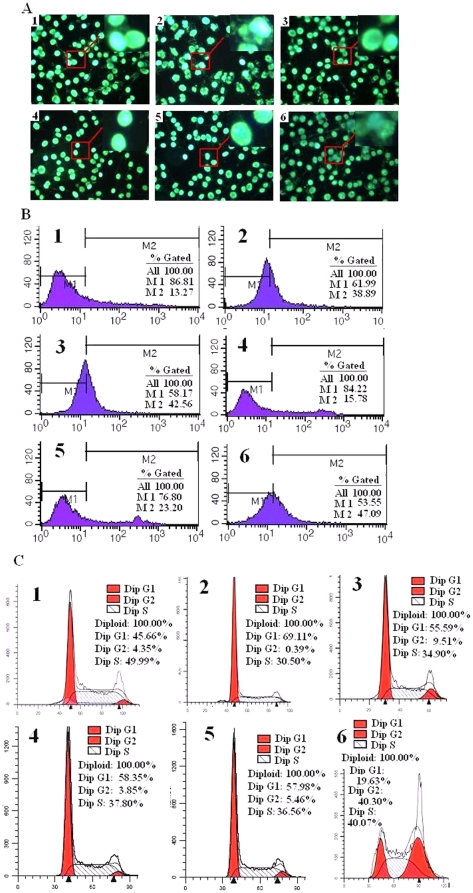
Morphological analyses of SGC-7901 cells. **A.** treatment with SPRC, SAC, PAG, PAG+SPRC and paclitaxel liposome (10 uM) in 24 h for determination of apoptotic changes analyzed under a fluorescence microscope. **B.** Analysis of apoptotic cells by Flow cytometric assay. **C.** Cell cycle distribution. Markers on pictures indicate: 1, Control. 2, SPRC 10 uM. 3. SAC 10 uM. 4. PAG 10 uM. 5. SPRC+PAG, each 10 uM. 6. Paclitaxel liposome 10 uM.

Apoptosis [21] is programmed cell death that is characterized by specific structural changes that include cell shrinkage, nuclear condensation and DNA fragmentation. Hoechst staining was used to observe the morphological changes of cells treated with Paclitaxel liposome, SPRC and SAC. As shown in Figure 2A, electron microscopy revealed that morphological changes characteristic of apoptosis occurred in SGC-7901 cells after treatment with SPRC ed for 24 hours. The cells exhibited a dense staining pattern with DNA fragmentation and absence of distinct nuclear membranes. Control cells did not exhibit similar morphological changes. Apoptosis-inducing effects of SPRC were assessed by flow cytometry (Figure 2B); 24 hour treatment of SGC-7901 cells with 10 uM SPRC resulted in a significant increase of about 26% of the apoptotic bodies when compared with the control group. In comparison, the percentage of apoptotic cells in Paclitaxel liposome and SAC-treated groups were less by about 8% and 4%, respectively.

The effect of SPRC on cell cycle activity of SGC-7901 cells was analyzed by performing propidium iodide staining followed by detection with flow cytometry. Consistent with its effect on cell growth inhibition, SPRC induced cell cycle arrest in SGC-7901 cells at G_1_/S phase (Figure 2C). 10 uM SPRC also resulted in an increased accumulation of 24% of cells in the G_1_ phase and reduced 20% of cells in the S-phase of the cell cycle, compared to the control group. This suggests that there is a blockage in the G_1_/S phase transition, which may cause cell growth suppression or apoptosis. The pro-apoptotic effect of SPRC as well as its effect of cell cycle arrest at G_1_/S phase was also largely inhibited by PAG.

### Effects of SPRC on CSE protein expression and activity and H_2_S levels

**Figure 3:**
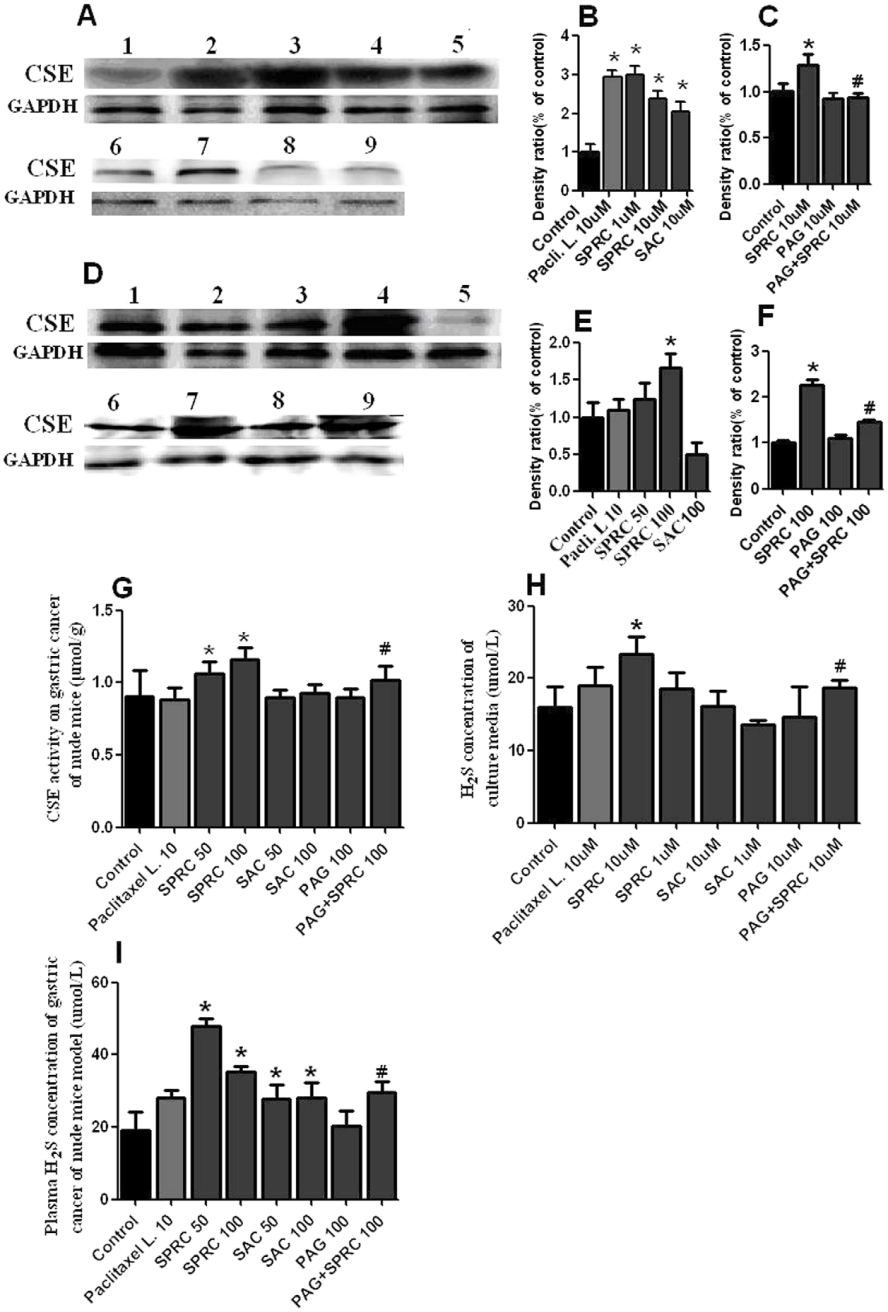
Effects of SPRC, SAC, PAG, PAG+SPRC and paclitaxel liposome on CSE protein expressions in SGC-7901 cells (A,B,C); gastric tumors of nude mice (D,E,F). For **A**, Lane 1, control group; Lane 2, paclitaxel liposome 10 uM; Lane 3, SPRC 1 uM; Lane 4, SPRC 10 uM; Lane 5, SAC 10 uM; Lane 6, Control group; Lane 7, SPRC 10 uM; Lane 8, PAG 10 uM; Lane 9, PAG+SPRC 10 uM. For **D**: Lane 1, control; Lane 2, paclitaxel liposome 10 mg/kg; Lane 3, SPRC 50 mg/kg; Lane 4, SPRC 100 mg/kg; Lane 5, SAC 100 mg/kg; Lane 6, control; Lane 7, SPRC 100 mg/kg; Lane 8, PAG 100 mg/kg; Lane 9, PAG+SPRC 100 mg/kg. Relative intensity is calculated by comparing with the intensity of GAPDH using densitometry (shown in the graphs on the right). * represent significant difference between control vs. SPRC, SAC and paclitaxel liposome treated groups (p<0.05). ^#^ represents significant difference between SPRC 10 uM vs. SPRC+PAG group. Figure **G** shows CSE activity (µmol/g) in the gastric cancer of all groups. Figure **H** shows H_2_S levels (µM) in cell culture media. Figure **I** show plasma H_2_S levels in gastric tumors of nude mice of different groups.

The expressions of CSE protein in SGC-7901 cells (Figures 3A, B and C) and tumors in nude mice (Figures 3 D, E and F) were significantly increased by SPRC treatment. SPRC at doses of 50 mg/kg and 100 mg/kg significantly increased CSE activity by about 1.4 fold (Figure 3G). CSE activity in tumors of SPRC-treated nude mice was higher than that in tissues of the SAC-treated group. H_2_S levels in cell culture media (Figure 3H), plasma of nude mice (Figure 3I) were measured. The H_2_S level in the cell culture media of 10 uM SPRC-treated cells and the plasma H_2_S of 50 mg/kg and 100 mg/kg SPRC-treated nude mice were also significantly increased compared with control group (P<0.05). When compared with the SAC-treated group of mice, the SPRC-treated group had significantly higher H_2_S level (P<0.05). The elevated levels of CSE protein expression, CSE activity and H_2_S in the SPRC-treated group were all largely reduced by PAG treatment. The results of these experiments thus suggested that the anticancer effect of SPRC was mediated by activation of the CSE/H_2_S pathway.

### Effects of SPRC and SAC on the gene expressions of Bax, p53 and Bcl-2

**Figure 4:**
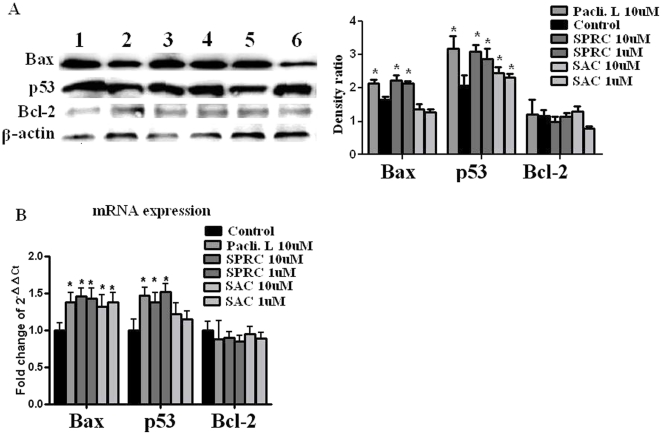
Effects of SPRC, SAC and paclitaxel liposome on protein expressions of Bax, p53 and Bcl-2 in SGC-7901 cells. **A.** Lane 1, paclitaxel liposome 10 uM; Lane 2, control; Lane 3, SPRC 10 uM; Lane 4, SPRC 1 uM; Lane 5, SAC 10 uM; Lane 6, SAC 1 uM. Relative intensity is calculated by comparing with the intensity of β-actin using densitometry (shown in the graphs on the right). **B.** Quantification of Bax, p53 and Bcl-2 mRNA expression. * represent significant significance between control vs SPRC, SAC and paclitaxel liposome at p<0.05 level.

The growth-suppression effect of SPRC on SGC-7901 cells was assessed to analyze the effects on cell cycle activity and expressions of the apoptosis regulator genes - Bax, p53 and Bcl-2. As shown in Figure 4A and 4B, we found that 24-hour SPRC treatment significantly increased the mRNA level (about 1.5 fold) and protein level of p53 and Bax of SGC-7901 cells.

### Growth suppression [22] and induction of cell apoptosis in tumors by SPRC

**Figure 5:**
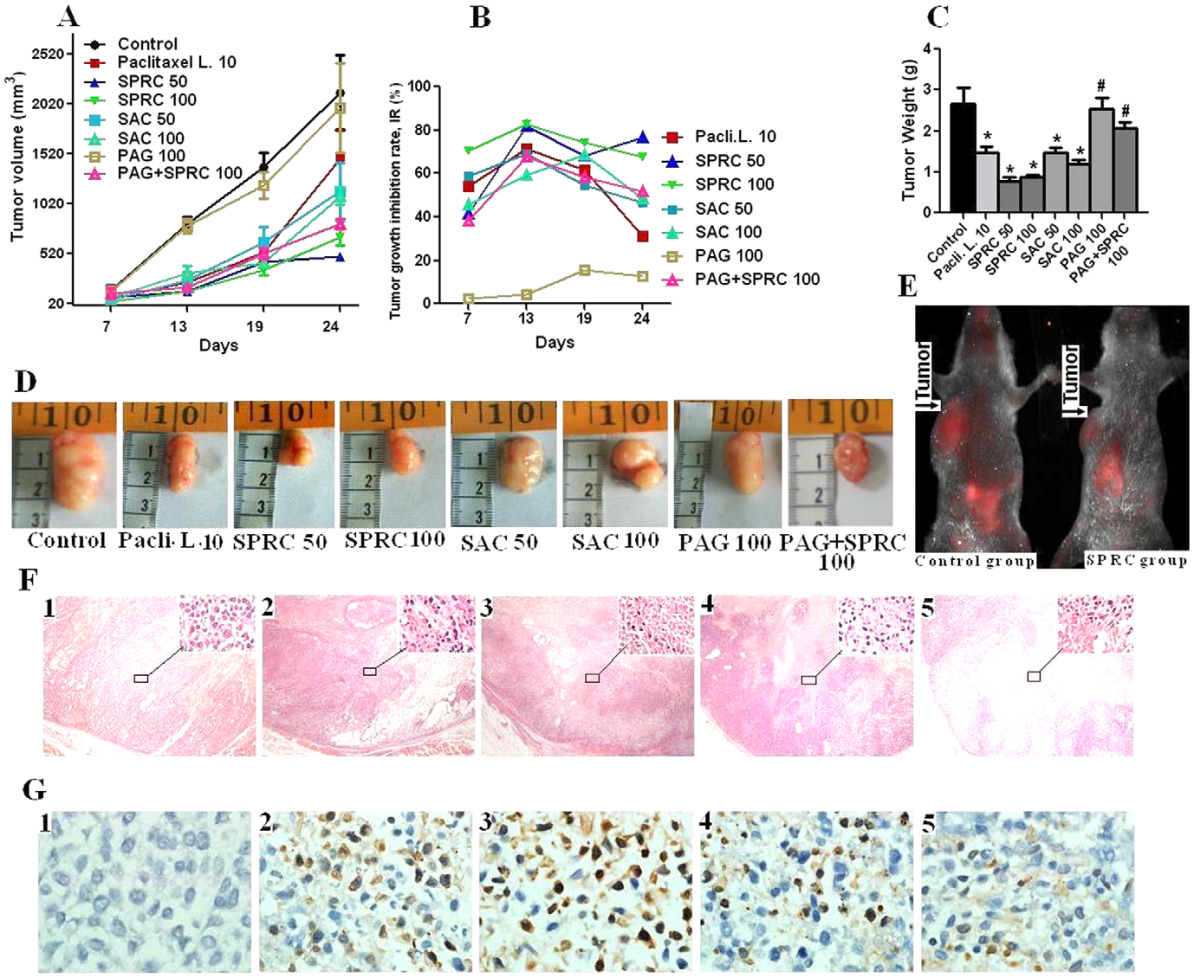
Effects of intra-peritoneal administration of SPRC, SAC and paclitaxel liposome on gastric cancer growth in male nude mice. **A.** Change in average tumor volume (mm^3^). **B.** Inhibition rate (IR) on tumor growth at different days. **C.** Change in tumor weight. **D.** Change in tumor size. **E.** *In vivo* tumor imaging of nude mice after treatment for 24 days. * represent significant difference (p<0.05) between control vs. SPRC, SAC and paclitaxel liposome treated groups. **^#^**represents significant difference (p<0.05) between SPRC 100 mg/kg vs. SPRC+PAG and PAG treated groups. **F.** H.E. staining with amplification of 4×10 and larger amplified pictures (100×10) at right corner. **G.** Tunnel staining (100×10) of apoptotic bodies of gastric tumors. Number markers on pictures indicate: 1, control group; 2, paclitaxel liposome 10 mg/kg group; 3, SPRC 50 mg/kg group; 4, SPRC 100 mg/kg group; 5, SAC 100 mg/kg group.

The development of gastric cancer evaluated by tumor volume was significantly reduced by SPRC treatment of 50 mg/kg and 100 mg/kg body weight every other day three times a week with an IR of 40–75% (Figure 5A and 5B). The tumor weight was also significantly reduced in SPRC-treated groups (Figures 5C) while PAG treatment significantly decreased the growth suppression function of SPRC. The representative tumors in different groups showed obvious tumor size change (Figures 5D and 5E).

We also examined whether the tumor growth inhibition by SPRC was reflected in apoptosis of tumor cells. H&E staining revealed (Figure 5F) more apoptotic tumor cell in paclitaxel liposome and SPRC-treated groups. TUNEL staining showed that the tumors from SPRC-treated mice had a markedly higher count of apoptotic bodies compared with the control tumors (Figure 5G). The increased incidence of apoptosis in the tumors was in conformity with the effect of tumor growth inhibition by SPRC, suggesting that SPRC exerted tumor growth inhibition partly by augmentation of apoptosis in the tumors.

### Effects of SPRC and SAC on the protein and mRNA expressions [23] of Bax, p53 and Bcl-2

**Figure 6:**
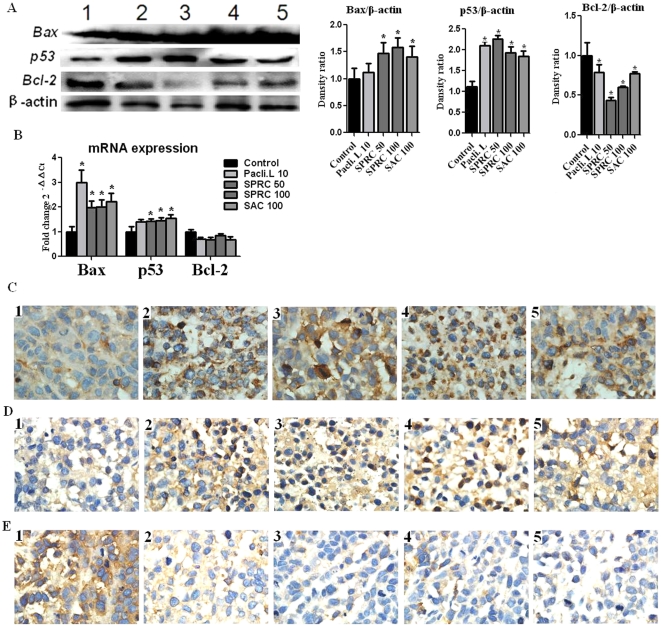
Effects of 24-day treatment with SPRC, SAC and paclitaxel liposome on Bax, p53 and Bcl-2 in gastric cancer-induced nude mice. **A.** protein expression, Lane 1 control; Lane 2 paclitaxel liposome 10 mg/kg; Lane 3 SPRC 50 mg/kg; Lane 4, SPRC 100 mg/kg; Lane 5, SAC 100 mg/kg. Relative intensity was calculated by comparing with the intensity of β-actin using densitometry. **B.** mRNA expression. *represent statistical significance between control vs SPRC, SAC and paclitaxel liposome- treated groups (p<0.05). **C.** Immunohistochemical staining of pro-apoptotic protein, Bax. **D.** Immunohistochemical staining of pro-apoptotic protein, p53. **E.** Immunohistochemical staining of pro-apoptotic protein, Bcl-2. Number markers on pictures in **Figure 6 C, D** and **E** indicate: 1, control group; 2, paclitaxel liposome 10 mg/kg; 3, SPRC 50 mg/kg; 4, SPRC 100 mg/kg; 5, SAC 100 mg/kg.

The suppression effect of SPRC on Gastric cancer was further assessed by Bax, p53 and Bcl-2. This effect of SPRC was also confirmed in gastric cancer-induced nude mice as shown in Figures 6A and 6B. The protein and mRNA levels of Bax and p53 in SPRC-treated tumors were significantly increased while those of Bcl-2 were decreased, compared with control tumors. The localization and amount of these three proteins in tumor tissues were also identified by immunostaining. As shown in Figures 6C and 6D, no obvious immunostaining of the pro-apoptotic proteins, Bax and p53, was observed in the control group while a strong signal of immunoreactivity was observed in SPRC- and Paclitaxel liposome-treated groups. A very weakly positive staining for the anti-apoptotic protein, Bcl-2, was detected in SPRC-, Paclitaxel liposome- and SAC-treated groups in Figure 6E. These results demonstrated that SPRC is able to regulate the gene expressions of Bax, p53 and Bcl-2 towards cell apoptosis.

### H_2_S demonstrated pro-apoptosis and anti- proliferation effect on SGC-7901 cells and gastric cancer model of nude mice

**Figure 7:**
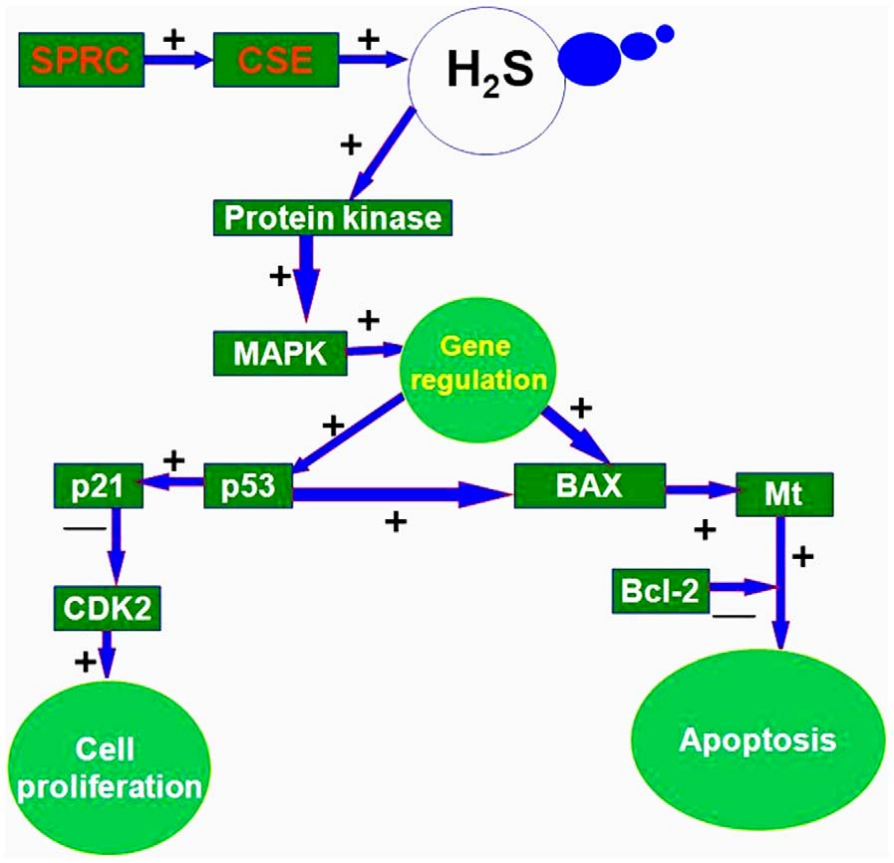
H_2_S demonstrated pro-apoptosis and anti- proliferation effect on SGC-7901 cells and gastric cancer model of nude mice: SPRC increased CSE activity and which is resulting in elevated H_2_S levels. The increase levels of H2S have turned modulates Bax, p53 and Bcl2 protein and mRNA expression. Bax has induced apoptosis through Mt and Bcl-2 expression. p53 leads to either inhibition of cell proliferation and DNA damage or apoptosis through Bax protein.

We found that SPRC increased CSE activity and which is resulting in elevated H_2_S levels, which in turn modulates gene expressions of Bax, p53 and Bcl2. Bax is directly induced mitochondria-driven apoptosis through increased Mt expression and decreased Bcl-2 expression. The p53 tumor suppressor protein plays a major role in cellular response to DNA damage and other genomic aberrations. Activation of p53 leads to either inhibition of cell proliferation and DNA damage or apoptosis through Bax protein in Figure 7. These results suggested a novel mechanism for the anticancer effects of SPRC via CSE/H_2_S-induced cell growth inhibition and apoptosis through the p53/Bax pathway.

## Discussion

In this study, we have demonstrated a novel anticancer effect of SPRC on gastric cancer and also provided data to suggest its possible mechanism. Several new points are generated from our study. Firstly, we demonstrated that SPRC was able to reduce cell growth ability significantly and also suppress the migration of SGC-7901 cells. Secondly, we found that SPRC was able to increase apoptosis-related morphological changes and induced a large increase in the percentage of apoptotic cells concurrently with an obvious cell cycle arrest at G_1_/S phase. Taken together, these two findings support evidence for an inhibitory effect of SPRC on SGC-7901 cells. Thirdly, we further demonstrated that intra-peritoneal administration of SPRC at doses of 50 and 100 mg/kg body weight every other day three times a week was effective in inhibiting the growth of gastric tumors in nude mice. Fourthly, we found that SPRC could increase CSE activity and also its protein expression resulting in elevated H_2_S levels in cell culture media and plasma of nude mice. Fifthly, we found that PAG, an inhibitor of CSE, could largely suppress the above functions of SPRC. Sixthly, we found that SPRC treatment caused obvious elevation of mRNA and protein expressions of Bax and p53.

It has been reported that H_2_S [24] [25] [26] can be generated in gastric cancer cells by the pyridoxal-5-phosphate-dependent enzyme CSE, for which L-cysteine is the substrate. CSE [27] could catalyze pyridoxal phosphate-dependent β-disulfide and elimination reaction, resulting in the formation of ammonium, pyruvate and thiocysteine. Thiocysteine then reacts with other thiols to form H_2_S. PAG has been used in several studies to test the biological effect of inhibiting endogenous H_2_S production [28] SPRC, an analog of SAC has the cysteine-containing structure which can undergo β-elimination and serve as a substrate of CSE. The key propargyl group in SPRC might combine with the activity sites of CSE to increase CSE activity. In our study, obvious increase in CSE protein expression in gastric cancer cells and tumors of nude mice was observed in the SPRC-treated group; this could be explained as a “compensatory effect” to produce H_2_S to cope with the apoptosis of cancer cells. Human lung fibroblasts treated by the H_2_S donor, NaHS, displayed an increase in DNA damage, cell cycle arrest and stabilization of p53, coupled with induction of downstream proteins such as p21, Bax and cytochrome c [29]. It has been reported that treatment of pancreatic acinar cells by H_2_S has been shown to induce apoptosis, resulting from the activation of caspase 3, decreased protein level of Bcl-2 and activation of Bax expression. H_2_S could also induce apoptosis of insulin-secreting beta cells by enhancing ER stress via p38 MAPK activation [30]. It was reported that p53 induces apoptosis by either increasing transcriptional activity of pro-apoptotic genes such as Bax or suppressing the activity of the anti-apoptotic gene of Bcl-2 family [31] [32]. Bax is also known to induce mitochondria-driven apoptosis. Here we also found that activation of CSE and increased H_2_S levels by SPRC treatment were coupled with elevated p53 and Bax expressions in gastric cells and tumors. These results suggested a novel mechanism for the anticancer effects of SPRC via CSE/H_2_S-induced cell growth inhibition and apoptosis through the p53/Bax pathway.

In conclusion, our *in vitro* and *in vivo* experimental results demonstrated that the hydrogen sulfide donor, SPRC, had obvious inhibitory and pro-apoptotic effects on gastric cancer. SPRC could increase CSE activity, resulting in an increased level of H_2_S, which in turn modulates gene expressions of Bax, p53 and Bcl2.

## Methods

All animal experimental protocols complied with the Animal Management Rules of local authorities and ‘Care and Use of the Laboratory Animals’ of the Experimental Animal Center of Fudan University, Shanghai, PRC.

### Details of the study approval by ethics committee

Animal Qualification Certificate No.: SCXK hu (Shanghai) 2009-0019

Study approval No.: Fudan University Experimental Animal Research Department, Approval No. SYXK hu (Shanghai) 2009-0082

Named review board: Chairman: Prof. Yi-zhun ZHU Dean, School of Pharmacy Fudan Univeristy. Vice Chairman: Prof. Weiyue Lu, Prof. Wei Wu, Prof. Xun Sun, Prof. Wenjiang Zhou

Members: Prof. Zhang yun yi, Prof. Li cong, prof. Jiang Chen, Prof. Yin geng li, Prof. Hong mei ji, Prof. Li xu yang.

Secretary: Tao lin lin

### Chemicals

SPRC was synthesized by reacting L-cysteine with propargyl bromide. The product was purified by re-crystallization from ethanol-water. The final product was verified by 1H nuclear magnetic resonance spectroscopy. SAC was purchased from Tokyo Kasei (Tokyo, Japan). Paclitaxel liposome was a gift from Luye Pharma Group Ltd., Singapore. Propargylglycine (PAG) was purchased from Yinzheng Chemical CO., LTD (Shanghai, China). MTT (dimethyl thiazolyl tetrazolium bromide) was purchased from AMRESCO Inc. USA. Hoechst staining, Cell Cycle and Apoptosis analysis Kits were purchased from Beyotime, China. RPMI 1640 medium were purchased from GIBCO™ Invitrogen, USA. Rabbit polyclonal antibodies to Bax, P53 and Bcl2 were purchased from Cell Signaling, USA and goat polyclonal antibody to CSE from Santa Cruz, USA. SuperScript II RT kit and SYBR Green PCR Master Mix were purchased from Takara, Japan. Tunnel staining kit was purchased from CALBIOCHEM, Germany.

### Cell line and culture

Human gastric carcinoma (SGC-7901) cells were obtained from Cell Bank of Shanghai Institute for Biological Sciences, China and cultured in RPMI 1640 medium with 10% fetal bovine serum (FBS), 100 U/mL penicillin and 100 µg/mL streptomycin in culture flasks at 37°C in humidified 5% CO_2_ incubator. The cells were fed until confluence and expanded by trypsinization and sub-cultured at lower numbers in new culture flasks.

### MTT assay

MTT (3-(4, 5-Dimethylthiazol-2-yl)-2, 5-diphenyltetrazolium bromide) assay was performed by using the method of Arumugam et al. [8] with slight modifications. Briefly, cells in suspension containing approximately 8,000 to 10,000 cells were added to each well of a 96-well culture plate and incubated for 24 h at 37°C in a humidified atmosphere of 95% air and 5% CO_2_. SPRC, SAC and Paclitaxel liposome were dissolved in culture medium and added to the cells in 96-well plates. 10 final concentrations of SPRC (1 uM, 10 uM, 100 uM, 1 mM, 5 mM, 10 mM, 15 mM, 20 mM, 25 mM and 30 mM), SAC (1 uM and 10 uM) and Paclitaxel liposome (10 uM) were added to the cells to study their effects on cell viability. The effect of combined treatment of 10 uM SPRC and 10 uM PAG was also studied. After 24 hours, 100 µL of 1 mg/ml MTT solution was added to each well and re-incubation followed for 4 hours. 100 µL DMSO was then added after removal of MTT solution and the plates were shaken for 10 minutes. The optical density of 96-well culture plates was measured using an enzyme-linked immunosorbent assay (ELISA) reader. The optical densities from the treated wells were converted to a percentage of living cells (“cell survival rate”) against the control using the following formula:
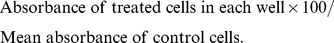



### Colony-forming assay

Colony forming experiment was performed according to the method of Wang et al. (1998, 2003) [9] [10]. The single cell suspension was produced and cultured in 12-well plates at a density of 150 cells per well. After 24 hours of planting, 2 concentrations of SPRC (10 uM, 1 uM) and SAC (10 uM, 1 uM) were added. An experiment with combined treatment of 10 uM SPRC and 10 uM PAG was also studied. Cells were incubated for 6 days. The cells were then fixed in 70% ethanol and stained with 1% Giemsa blue. The colonies that consisted of >50 cells were scored and compared with the normal control group. Each experiment was repeated three times.

### Hoechst staining assay

Apoptosis [11] [12] was determined using a Hoechst Staining Kit (Beyotime). SGC-7901 cells were treated with SPRC (10 uM), SAC (10 Um), Paclitaxel liposome (10 uM) and combined SPRC and PAG (each 10 uM) for 24 hours. The cells were rinsed twice in PBS and then fixed for 10 minutes at 4°C and then stained by karyophilic dye, Hoechst 33258, for 5 minutes. After a final rinse in PBS, the cells were mounted in moiwol, an anti-fade agent, and visualized under ultraviolet light with a fluorescence microscope. Because this dye stains both apoptotic and non-apoptotic cells, apoptotic cells were identified as those displaying chromatin condensation and nuclear fragmentation.

### Flow cytometric analysis of cell cycle phase distribution and detection of apoptosis

All cells were first plated at a density of 2.5×10^5^ cells/well in 6-well plates. After incubating with SPRC, SAC, Paclitaxel liposome and combined SPRC and PAG treatment for 24 hours, the cells were detached and collected into flow cytometry tubes and centrifuged at 1000 rpm for 5 min to obtain a cell pellet.

### Apoptosis assay

The cells were stained using the cell apoptosis detection Kit (Beyotime) according to the manufacturer's instructions. The cell pellet was washed twice with PBS. 1× Binding buffer was added to 1×10^6^ cells/ml, and PI was then added in the dark. After incubation for 30 min in the dark, the cells were immediately analyzed using Cyan flow cytometer (BD FACSCalibur) and ModFit software.

### Cell cycle analysis

Cell cycle analysis was performed by the method of Herman-Antosiewicz et al [13]. Briefly, cells were incubated in culture media alone and culture media containing SPRC, SAC and Paclitaxel liposome (10 uM) at 37°C for 24 h. Cells were washed by cold PBS, fixed in 70% ethanol, and stored at 4°C for subsequent cell cycle analysis. Fixed cells were washed with PBS once, then re-suspended in 1 mL of PI staining reagent (50 mg/ml propidium iodide and 1 mg/ml RNAse in 1 ml of sodium citrate buffer, pH 7.4). Samples were incubated in the dark for 30 min before cell cycle analysis. The distribution of cells in the cell cycle was measured by Cyan flow cytometer (BD FACSCalibur) analysis system and quantitation of cell cycle distribution was performed using Multi-cycle Software (ModFit software). The percentage of cells in G1, S and G2 phases were calculated.

### Wound healing assay

Cells were seeded into 6-well culture plates and allowed to grow to 90% confluence. Similar sized wounds were introduced to monolayer cells using sterile pipette tips. Wounded monolayer cells were washed 3 times by PBS to remove cell debris; SPRC and SAC were then added at two concentrations (1 uM, 10 uM) and paclitaxel liposome (10 uM). The speed of wound closure was monitored and photographed after 12 and 24 hours. The comparative speed of wound closure of treated cells against the control was calculated using the following formula: (the wound width of treated cells at 0 hour - the wound width at 24 hours)/(the wound width of Control cells at 0 hour - the wound width at 24 hours)×100.

### Quantitative real-time PCR

Total RNA was reverse transcribed using a SuperScript II RT kit (Takara, Japan) primed with oligo (dT). Expressions of Bcl2, Bax and P53 mRNA were examined by real-time PCR with Step One PCR system (Applied Biosystems). 2 ul of reverse transcribed cDNA product was added in a 20 ul reaction mixture containing 10 ul SYBR Premix EX Taq (2×), 0.4 ul ROX Reference Dye (50×), 0.4 uM forward primer and 0.4 uM reward primer. The cycling conditions were as follows: pre-incubation at 95°C for 30 s, followed by 40 cycles of denaturation at 95°C for 5 s, annealing and extension at 60°C for 31 s. At the completion of cycling, melting curve analysis was performed to establish the specificity of the PCR product. The relative quantitative value was expressed by the ΔΔC_T_ method. Control was used as the reference sample and β-actin was used as the endogenous control. Each experiment was performed in duplicate and repeated three times. The primer sequences and expected product size were as follows: Human β-actin: forward, 5′-CGTGGACATCCGCAAAG-3′ and reverse, 5′-TGGAAGGTGGACAGCGA-3′ (201 bp); human Bax: forward, 5′-ATGCGTCCACCAAGAAGC-3′ and reverse, 5′-GTCCACGGCGGCAATCA-3′ (95 bp); human bcl2: forward, 5′-AACTGGGGGAGGATTGTG-3′ and reverse, 5′-AGGTGCCGGTTCAGGTAC-3′ (128 bp); human p53: forward, 5′-ACCACCATCCACTACAACTA-3′ and reverse, 5′-AAACACGCACCTCAAAGC-3′ (136 bp).

### Western blot analysis

Cells were plated to dishes and treated with SPRC (1 uM, 10 uM), SAC (1 uM, 10 uM) and Paclitaxel liposome (10 uM) for 24 hours. After 24 hours of treatment, the cells were washed twice with cold PBS. The cells from each sample were solubilized in RIPA buffer (50 mM Tris-HCl (pH 8.0), 150 mM NaCl, 1% NP40, 0.1% SDS, 10 mM sodium deoxycholate, 1 mM phenylmethyl-sulfonylfluoride) and kept on ice for 30 minutes. Cells lysates were centrifuged at 10,000 g at 4°C for 10 min and the supernatants were stored at −70°C until assay. Protein concentration was measured by the Bradford method. 25 *µl* protein for each group mixed with 5 *µl* loading buffer were separated by 10% SDS-polyacrylamine gel and transferred to polyvinylidene fluoride membranes [14]. The membranes were blocked for 2 hours in 5% skim milk powder in Tris-buffer saline containing 0.05% Tween 20 (TBST) at room temperature. The resulting blots were then probed with polyclonal CHT (cystathionine γ-lyase)/CSE Ab (1∶500, Santa Cruz, USA), polyclonal Bcl-2 Ab (1 ∶ 1000, Cell Signaling, USA), polyclonal Bax Ab (1 ∶ 1000, Cell Signaling, USA) and polyclonal p53 Ab (1 ∶ 500, Cell Signaling, USA) for overnight at 4°C. Membranes were washed for three times with TBST for 10 minutes each and incubated for 2 h with the anti-rabbit (for Bax, p53 and Bcl-2 Ab) and anti-goat (for CSE Ab) secondary antibody at room temperature (1∶ 2000, Santa Cruz, CA), again followed by three times washing with TBST for 10 minutes each. The immunoreactive proteins were detected using the ECL Western blotting detection system (Alpha Imaging Inc., USA), according to the manufacturer's instructions. To measure the expression of each gene, the relative intensity was calculated by comparing with the intensity of β-actin (for Bax, p53 and Bcl-2) and GAPDH (for CSE) using densitometry.

### Measurement of human and mouse plasma H_2_S concentration

To measure H_2_S concentration, 500 µL of culture media from each group (n = 6, 1×10^6^ cells per sample), 75 µL of plasma of nude mice (n = 6) and 75 µL of plasma of gastric cancer patients (n = 3) and 425 µL of distilled water were added to a micro-tube containing zinc acetate (1% w/v; 250 µL). Subsequently, *N,N*-dimethyl-*p*-phenylenediamine sulphate (NNDPD, 20 µM; 133 µL) in 7.2 M HCl was added, then followed by FeCl_3_ (30 µM; 133 µL) in 1.2 M HCl. Trichloroacetic acid (TCA, 10% w/v; 250 µL) was then used to participate any protein. This optical absorbance of the resulting solution was measured at 670 nm using a 96-well microplate reader (Tecan Systems Inc., Switzerland).

### Establishment of human gastric cancer model in nude mice

Male nude mice, aged 3–4 weeks, weighed 18–20 g, were purchased from Animal Experiment Center (Shanghai Institute for Biological Sciences, China) and bred in specific pathogen-free (SPF) conditions, housed in mesh cages under controlled conditions of temperature (23±3°C) and relative humidity (50±20%), with 10–15 air changes per hour and light illumination for 12 hours a day. The animals were allowed access to food and purified water *ad lib*.

Injections of 200 µL (1×10^7^ SGC-7901 cells) per site were made into the flanks of nude mice to establish a model of tumor-bearing mice [15] [16]. About 25 days post-implant, the bearing-tumors were extracted. After necrotic tissue and non-cancerous tissues of the specimens were removed, the remaining cancerous tissues were cut into small pieces of about 1 mm^3^ in size. The gastric tumor models were established by implanting tumor bits directly to the flanks of nude mice. 7 days post-implant, when the diameter of the tumor had reached about 0.3–0.5 cm, the tumor-induced nude mice were divided into a control group (n = 5), paclitaxel liposome (10 mg/kg)-treated group (n = 5), SPRC (50 mg/kg and 100 mg/kg)-treated groups (each n = 8), SAC (50 mg/kg and 100 mg/kg)-treated groups (each n = 8) and combined treatment group of SPRC (100 mg/kg)+PAG (100 mg/kg) (n = 8). All the mice were injected intra-peritoneal every other day with 100 ul of the respective drugs in the different treatment groups while sterile water was injected into the control group.

### General observation and tumor measurements

The general condition of the mice was observed every day. Nude mice were weighed and the sizes of tumors were measured every 7 days. The size of each tumors was first measured for its length (L) and width (W); the volume was then calculated, using the formula V = W^2^×L×0.5, where L is the longest diameter and W the shortest width. The tumor growth inhibition rate (IR) was calculated according to the volume of the tumor: IR (%) = (1−volume of treated group/volume of control group)×100%. The experiments were terminated and the mice sacrificed by cervical decapitation 24 days after treatment. The tumors harvested at autopsy [17] were weighed and sectioned, and fresh tissue samples were wrapped in aluminum foil and immediately frozen in liquid nitrogen [18]. All animal procedures were performed according to standard protocol and in accordance with the standard recommendations for the proper care and use of laboratory animals.

### H.E and TUNEL staining

Cell apoptosis and necrosis were examined by H.E and Tunnel staining. The frozen samples of gastric tumor growth were cut into 5 µm-thick sections, mounted on 3-aminopropyltriethoxysilane (APES)-treated slides. Slides were fixed with 4% paraformaldehyde solution. The slides were observed and imaged by an Olympus DP72 microscopic camera and analyzed by Image Pro 7 software.

### Real time PCR, western blot and immunohistochemistry analysis

We analyzed the protein level of CSE, Bax, p53 and BCL-2 in tumor tissues (western blot methods [19] as described in protocol 1). For immunohistochemistry staining, the slides of tumor tissue were blocked by ass serum for 1 h at room temperature and incubated with rabbit anti-Bax, anti-p53, and anti-Bcl2 (1∶1000, cell signal) overnight at 4°C, then incubated with anti-rabbit secondary antigen for 1 h at room temperature and stained. Between each step, the slides were washed 3× with PBS. The slides were observed and imaged by an Olympus DP72 microscopic camera and analyzed by Image Pro 7 software. mRNA levels of Bax, p53 and Bcl-2 were also evaluated by realtime-PCR (methods, primer sequence and expected products size were the same as in protocol 1).

### Measurement of CSE activity

CSE activity was assayed as described previously. Briefly, 0.1 g of tumor tissues (n = 6 per group) was thawed on ice and homogenized in 2 mL of 100 mM ice-cold potassium phosphate buffer (pH 7.4). Tissues homogenates were centrifuged at 12,000 g for 10 minutes at 4°C, and the supernatant was then used for the assay. The reaction mixture contained 20 µL of 10 mM L-cysteine, 20 µL of 2 mM pyridoxal-5-phosphate, 30 µL of PBS, and 430 µL of tissue homogenate. The catalytic reaction was initiated by transferring the reaction mixture from ice to a 37°C water bath for 30 minutes. Then 250 µL of 1% zinc acetate was added to the tubes using a syringe to trap the evolved H_2_S. Next, 250 µL of 10% TCA was added to quench the enzymatic reaction. Finally, 133 µL of NNDPD in 7.2 M HCl and 133 µL of FeCl_3_ in 1.2 M HCl were added. The absorbance of the final reaction mixture was measured at 670 nm using a 96-well microplate reader (Tecan System Inc., Switzerland). All samples were assayed in duplicate, and the H_2_S concentration for each sample was calculated against a calibration curve using NaHS (3.125 µM −250 µM). Results were expressed as µmol/g. Protein content was determined by using a BCA assay kit (Beytime Biotechnology, China).

### Statistical analysis

All values were presented as means and standard deviations. One-way analysis of variance (ANOVA) was used to examine statistical comparisons between groups. The statistical significance of difference between two groups was determined using two-tailed Student's *t*-test [20]. A probability value of <0.05 was taken to indicate statistical significance. All analyses were performed using SPSS 12.0.
